# Quality Evaluation, Storage Stability, and Sensory Characteristics of Wheat Noodles Incorporated with Isomaltodextrin

**DOI:** 10.3390/plants10030578

**Published:** 2021-03-18

**Authors:** Da-Wei Huang, Yung-Jia Chan, Yuan-Chao Huang, Ya-Ju Chang, Jen-Chieh Tsai, Amanda Tresiliana Mulio, Zong-Ru Wu, Ya-Wen Hou, Wen-Chien Lu, Po-Hsien Li

**Affiliations:** 1 Department of Biotechnology and Food Technology, Southern Taiwan University of Science and Technology, Tainan City 710, Taiwan; hdw0906@stust.edu.tw; 2 College of Biotechnology and Bioresources, Da-Yeh University, Dacun, Changhua 51591, Taiwan; d0867601@cloud.dyu.edu.tw; 3 SPharma Power Biotec Co., LTD., Taipei City 105412, Taiwan; snack997@ppbiotec.com.tw (Y.-C.H.); car-rie@ppbiotec.com.tw (Y.-J.C.); 4 Department of Medicinal Botanical and Health Applications, Da-Yeh University, Dacun, Changhua 51591, Taiwan; jenchieh@mail.dyu.edu.tw (J.-C.T.); tresiliana@gmail.com (A.T.M.); ian44989@gmail.com (Z.-R.W.); 5 Seafood technology division, Fisheries Research Institute, Council of Agriculture, Keelung 202008, Taiwan; ywhou@mail.tfrin.gov.tw; 6 Department of Food and Beverage Management, Chung-Jen Junior College of Nursing, Health Sciences and Management, Hung-Mao-Pi, Chia-Yi City 60077, Taiwan

**Keywords:** wheat noodles, isomaltodextrin, storage stability, cooking quality, sensory evaluation, microstructure

## Abstract

Wheat noodles incorporated with isomaltodextrin were assessed in relation to physicochemical properties (color), microstructure features, biochemical composition (fiber profile), cooking properties, textural attributes, and sensory evaluations during different storage temperatures (25, 4, −20 °C) and periods (0, 3, 6, 9, 12, 15, 18, 21, 24 months). Meanwhile, an accelerated study was also carried out at 40 °C storage conditions for 12 months to evaluate the fiber profile changes. Under different conditions, the overall quality of both raw and cooked noodle samples depended slightly on both the type and amount of added fiber isomaltodextrin, resistant starch (RS), insoluble high-molecular-weight dietary fiber (IHMWDF), and soluble high-molecular-weight dietary fiber (SHMWDF). However, this significantly changed for the fiber profile under 40 °C of storage for 12 months. Cooking quality, fiber profile, and color parameter did not differ by storage at −20 °C after 24 months than at 0 months, and noodles only slightly differed in texture and sensory characteristics. On sensory analysis, noodle samples were acceptable by panelists, with an acceptability score >5. In short, storage temperature is one of the most important factors in preserving food stability and retail properties. Isomaltodextrin noodles samples should be stored at low temperature to preserve the product functionality.

## 1. Introduction

The glycemic index (GI), which is based on glycemic response, is a well-established indicator of the increase in blood glucose potential of a carbohydrate food [1]. Low GI diets play a vital role in the management and control of diabetes [2]. Previous research reported that the probable effects of a low-GI diet are lowering insulin secretion in type 2 diabetes and decreasing daily insulin requirements in type 1 diabetes [3]. Higher consumption of water-soluble dietary fiber, such as isomaltodextrin, above the level recommended by the American Diabetes Association (ADA) enhances glycemic control, reduces hyperinsulinemia, and lowers plasma lipid concentrations in people with type 2 diabetes [4]. However, noodles that are primarily formulated with wheat flour are not suitable for consumption by people with diabetes because of the high GI effects, which are evaluated for glycemic response (higher digestion rate or glucose absorption rate in the small intestine) [5,6].

In 2016, isomaltodextrin was assessed as a food that is generally recognized as safe (GRAS) by the US Food and Drug Administration (FDA) [7]. Isomaltodextrin presented health effects such as inhibition of fat absorption [8], anti-inflammatory properties, reduced risk of developing insulin resistance and associated metabolic diseases [9], diminished postprandial blood glucose [10], positive effects on intestinal flora [11], and inhibition of hen egg ovalbumin allergic response by inducing immune tolerance in mice [12]. Isomaltodextrin, a low-viscosity, water-soluble dietary fiber, which is enzymatically produced from corn starch, is also useful in reducing insulin secretion due to the reduction in sugar absorption by inhibiting the disaccharidase-related transport system in both rats and healthy humans [13]. Furthermore, isomaltodextrin improved glucose tolerance after sucrose, maltose, and maltodextrin loading and also greatly lowered the accumulation of body fat, regardless of changes in body weight in male Sprague–Dawley rats [14]. Hence, because typical noodles will simply increase blood glucose level, fortification of low-GI ingredients (isomaltodextrin) in noodles may benefit people with diabetes and allow then to enjoy noodles as part of a healthy diet. However, few studies have investigated the stability and physicochemical of isomaltodextrin under different storage conditions.

Storage stability studies of food products is a vital characteristic for both manufacturers and consumers, particularly in terms of food safety. Additionally important are physical and chemical features, appearances, and sensorial properties (organoleptic). Storage stability studies can provide important information to manufacturers and consumers to certify a high-quality product throughout the storage period [15]. Meanwhile, the products must be acceptable for consumers and comply with the nutritional value on the labeling. Choosing suitable storage-stability study models and data-analysis techniques are important to anticipate the shelf life, consider the changeability in environmental conditions, and guarantee real-time monitoring of products [16]. Appropriate scientific estimation and calculation of the storage stability of food commodities maintains the safety and quality of the products and can prevent food waste during the distribution and consumption stages of the food chain [17].

Therefore, this study aimed to investigate and evaluate the storage stability study of wheat noodles fortified with isomaltodextrin, in terms of physicochemical characteristics, microstructure features, dietary fiber profile, cooking quality, and sensory attributes during different storage temperature and periods. Meanwhile, it is hypothesized that isomaltodextrin wheat noodles along the 24 months of storage at low temperature, which is 4 and −20 °C, with only minor alterations in texture and sensory quality.

## 2. Materials and Methods

All chemical and reagents used in this study were purchased from Sigma-Aldrich Chemical (St. Louis, MO, USA) (ACS certified grade). Total dietary fiber assay kit (K-TDFR) was provided by Megazyme (Wicklow, Ireland). Isomaltodextrin (GRAS no. 610) was purchased from the Hayashibara (Okayama, Japan). All samples were established and analyzed in triplicate. 

### 2.1. Noodles Sample

Wheat flour composed of hard red spring wheat and hard red winter wheat in a 2:3 proportion was provided by Chi-Fa Enterprise (Taichung, Taiwan). The analysis of moisture (Method 934.01), crude protein (Method 984.13), and ash content of corms followed the methods of the Association of Official Agricultural Chemists (1990). The moisture, crude protein, and ash content of wheat flour was 13.52, 11.74, and 0.35%, respectively. Isomaltodextrin was blended by using a domestic blender and passed through a 100-mesh (150 μm) sieve. Wheat noodles were prepared as described [18] with modification. The formulated flours were prepared (5% of isomaltodextrin), and 2% salt was added; this mixture was mixed with water at low speeds for 3 min then rolled into a 2 mm thick sheet of dough. Folding and sheeting were repeated twice more. The dough sheet could rest for 30 min then was put through the sheeting rolls 3 times at progressively decreasing roll gaps of 2.60, 2.33, and 2.00 mm. From this dough sheet, the noodle strands were cut into strands of 18.1 × 0.12 × 0.11 cm, and the prepared noodles were dried in an oven at 55 °C for 12 h. Next, the dried noodle samples were packed in high-density polyethylene (HDPE) bags and stored in a dry box until further analysis use.

### 2.2. Storage Stability Study

To understand the effect of storage conditions on the product quality, and by referring the method of ICH Q1A (R2) Stability Testing of New Drug Substances and Products (European Medicines Agency), the noodle samples were stored under four different conditions, which is in general ambient condition (25 °C ± 2 °C/60% RH ± 5% RH), refrigerated storage temperature (5 °C ± 3 °C), freezing temperature (−20 °C ± 5 °C) for 24 months, and accelerated (40 °C ± 2 °C/75% RH ± 5% RH) conditions for 12 months (Figure S1). The noodle samples, packed in HDPE bags and stored under these conditions, were analyzed for physicochemical properties, such as cooking quality, color characteristics, texture analysis; changes in isomaltodextrin, resistant starch (RS), insoluble high-molecular-weight dietary fiber (IHMWDF), soluble high-molecular-weight dietary fiber (SHMWDF), sensory characteristics, as well as microstructure properties by scanning electron microscope (SEM). The noodle samples placed under different conditions were withdrawn at 3-month intervals for the above analyses. Still, the analyses were completed within 5 days of every single withdrawal, and the analyses were carried out in three replications.

### 2.3. Cooking Quality

The impact of storage conditions on the cooking characteristics of noodle samples was analyzed by the method of the American Association for Clinical Chemistry (AACC) (66–50) (AACC 2000). Cooking time (min) of the noodle samples was determined by starting the timer when adding one sample of 25 g to a beaker containing 300 mL boiling distilled water. The sample was stirred to ensure that noodles were separated. Cooking time corresponded to the disappearance of the opaque center core of the noodle when the noodle was squeezed between two clear glass plates. Cooking loss (%) was examined by drying the cooking water in an oven at 105 °C until constant weight was achieved. Cooking loss was calculated as follows:

(1) $$\mathrm{Cooking}\;\mathrm{loss}\;(\%)\;=\;\frac{\mathrm{weight}\;\mathrm{of}\;\mathrm{dried}\;\mathrm{residues}\;\mathrm{in}\;\mathrm{noodles}\;\mathrm{cooking}\;\mathrm{water}\;\left(g\right)}{\mathrm{weight}\;\mathrm{of}\;\mathrm{uncooked}\;\mathrm{noodles}\;\left(g\right)}\;\times 100$$

### 2.4. Dietary Fiber Profile Analysis

#### 2.4.1. Determining Resistant Starch (RS)

The RS of noodle samples was analyzed by using the Megazyme kit (Megazyme K-TDFR, Wicklow, Ireland), which recognizes the method of the Association of Official Analytical Chemists (AOAC) (Method 2002.02/AACC Method 32-40.01). 

#### 2.4.2. Determining Isomaltodextrin

In this study, the content of isomaltodextrin usually was based on AOAC Method 991.43 “Total, Soluble, and Insoluble Dietary Fiber in Foods” (First Action 1991) and AACC Method 32-07.01 “Determination of Soluble, Insoluble, and Total Dietary Fiber in Foods and Food Products” (Final Approval 10-16-91). 

#### 2.4.3. Determining IHMWDF and SHMWDF

Analysis of IHMWDF was based on the AOAC Official Method 2011.25 “Insoluble, Soluble, and Total Dietary Fiber in Foods” (First Action 2011). 

### 2.5. Color Measurement

Color of noodle samples was measured with a ZE2000 Color Measurement colorimeter (Nippon Denshoku) by using the CIE L*a*b* system. Color parameter was reported as lightness (L*); positive a* represents the redness, and negative a* is green; positive b* is yellowness, and negative b* is blue. A standard black and white ceramic tile was used to calibrate the instrument before every measurement. Color measurements were performed at room temperature in pentaplicates for every test sample.

### 2.6. Texture Profile Analysis

Texture analyzer model Brookfield CT3 Texture Analyzer (AMK CT3) specific for the food industry was used to measure noodle texture. Five noodle strands were arranged adjacent to each other on the fixture base tables and tested for hardness (g), adhesiveness (g.s), maximum tensile strength (g), and tensile fracture distance (mm) under the experimental conditions of 250 kg load cell and 10 mm/s cross head speed. The analyses were carried out with three repetitions and five replicates.

### 2.7. Sensory Evaluation

A total of 30 panelists (15 females and 15 males), aged from 25–35 years old, who were students from the College of Biotechnology and Bioresources, were randomly selected to participate in this sensory evaluation session. The sensory evaluation test was conducted in a sensory laboratory at room temperature and strictly followed the GB/T 13662-2008 and ISO 4121 criteria. 

### 2.8. Microstructural Characteristics

The noodle samples stored under three different conditions 25, 4, −20 °C for 3, 12, and 24 months were coated with gold-palladium (Model JBS-ES 150, Ion sputter coater, Topon Corp., Japan), and the instrument was used with a model of the ABT-150S system (Topon Corp., Kyoto, Japan) equipped with an Olympus BX53 polarized light microscope (PLM) and a CCD camera to detect the collaboration and effects of the noodles matrix and variations during the storage study.

### 2.9. Statistical Analysis

Experimental assessments and analysis were carried out in triplicate. Data were reported as a mean score for each individual attribute. For statistical analysis, all data were assessed by using single-factor ANOVA. If the F-value was significant (*p* < 0.05) on ANOVA, then Duncan’s new multiple range tests was used to correlate treatment means.

## 3. Results and Discussions

### 3.1. Effects of Storage on Cooking Quality

Cooking quality and characteristics of wheat noodles fortified with isomaltodextrin during storage periods of 0 to 24 months, at 25, 4, and −20 °C are summarized in Table 1. Determining the cooking quality aimed to understand the interrelationship between storage temperature and storage time in terms of noodle quality, to ensure consumer acceptancy and the mouthfeel of the products. The cooked weight gain of the noodles stored at 4 and −20 °C did not significantly differ from 0 to 24 months. However, the cooked weight gain significantly differed after 18 months for noodles stored at 25 °C (62.62 ± 0.15, 62.56 ± 0.16, 62.37 ± 0.16 g for 18, 21, and 24 months, respectively) as compared with 0 months (63.97 ± 0.60 g). The results agreed with a previous study [19], finding that the reduction in cooked weight was probably due to an increase in cooking loss. Cooking loss was to measure the number of solids remaining in the cooking water, which was caused by the leaking of amylose and solubilization of soluble proteins [20]. The cooking loss for noodles stored at 25 °C significantly differed from 3.71 ± 0.02 to 3.93 ± 0.04, 3.98 ± 0.05, 4.08 ± 0.07, and 4.13 ± 0.07 at 0, 15, 21, and 24 months. Appropriate cooking time and adequate cooking water may control the cooking loss, which deeply affects the texture and sensory attributes of the noodles.

### 3.2. Effects of Storage on Fiber Profile

**Figure 1:**
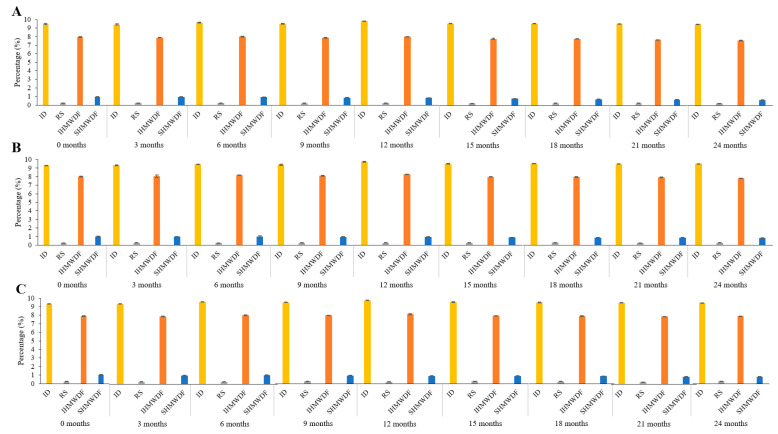
Fiber profile of wheat noodles fortified with isomaltodextrin during different storage period (0, 3, 6, 9, 12, 15, 18, 21, and 24 months) at 25 °C (**A**), 4 °C (**B**), and −20 °C (**C**). ID, isomaltodextrin; RS, resistant starch; IHMWDF, insoluble high-molecular-weight dietary fiber; SHMWDF, soluble high-molecular-weight dietary fiber.

**Figure 2:**
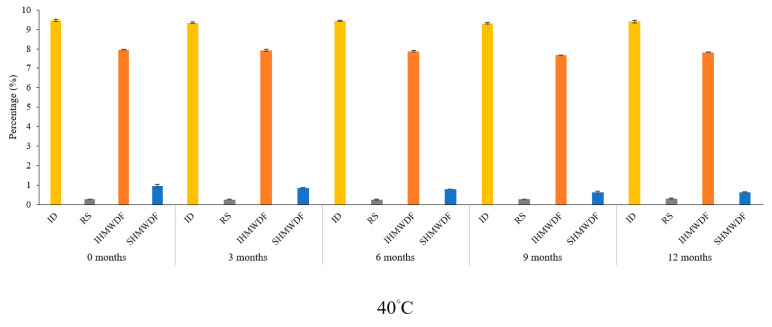
Fiber profile of wheat noodles fortified with isomaltodextrin during different storage periods (0, 3, 6, 9, and 12 months) at 40 °C storage. ID, isomaltodextrin; RS, resistant starch; IHMWDF, insoluble high-molecular-weight dietary fiber; SHMWDF, soluble high-molecular-weight dietary fiber.

Because of different structural characteristics of fibers, molecular weight distribution and spatial density of different dietary fibers added to noodles compounds can have positive or negative effects on noodles properties [21]. Therefore, assurances and preserved product quality control have become more crucial and have led to the need to determine the amount of dietary fiber composition in final products. Figure 1 illustrates the fiber profile of wheat noodles fortified with isomaltodextrin during the different storage periods at 25, 4, and −20 °C storage. Figure 2 shows the fiber profile of wheat noodles fortified with isomaltodextrin during the different storage periods at 40 °C storage. The isomaltodextrin content ranged from 9.47 ± 0.06 to 9.31 ± 0.03%, RS content from 0.24 ± 0.01 to 0.22 ± 0.02%, IHMWDF content from 7.96 ± 0.04 to 7.44 ± 0.04%, and SHMWDF content from 0.95 ± 0.02 to 0.61 ± 0.02%, from 0 to 24 months of storage at 25 °C (Table S1). However, storage at 4 °C and −20 °C slowed the degradation of SHMWDF, which was 1.01 ± 0.05 to 0.79 ± 0.03% and 1.01 ± 0.04 to 0.80 ± 0.03%, respectively, from 0 to 24 months of storage (Tables S2 and S3). The composition of isomaltodextrin, RS, and IHMWDF did not differ by storage and storage temperature. Regardless, SHMWDF content was degraded with storage at ambient temperature. Previous study has revealed that dietary fibers with high molecular weight have stronger viscosity than those with low molecular weight [22]. Moreover, high-molecular-weight dietary fiber, including cellulose, RS, and guar gum, originally from wheat flour, had a better effect on noodles than low-molecular-weight dietary fiber (Table S4) [23].

Dietary fiber is proclaimed to help avoid obesity caused by high-fat diets [24], by reducing energy intake, increasing the viscosity of intestinal contents, delaying gastric exhaustion, and augmenting the short-chain fatty acid content in the large bowel, thus preventing central appetite and encouraging the release of anorexic intestinal hormones [25]. Meanwhile, dietary fiber as a food additive helps to maintain the level of fiber content in food and encourage the technological functionalities of food, such as gelatinization, product consistency, colloidal stability, texture, and viscosity [26]. As compared with insoluble dietary fiber, soluble dietary fiber is more common because of its critical physiological roles and benefits in physical and chemical properties [27], for example, the release of hyperlipidemia, controlling diabetes mellitus, effect on cardiovascular disease, and monitoring of colon cancer [28]. Because of the characteristics of water retention, gel formation, fat simulation, and thickening effects, the addition of soluble dietary fiber can significantly enhance the texture, shelf life, stability, and sensory properties of starch-based food; decrease the rate of aging; preserve the relative stability of the rheological and tissue properties [29].

### 3.3. Effects of Storage on Color Parameters

**Figure 3:**
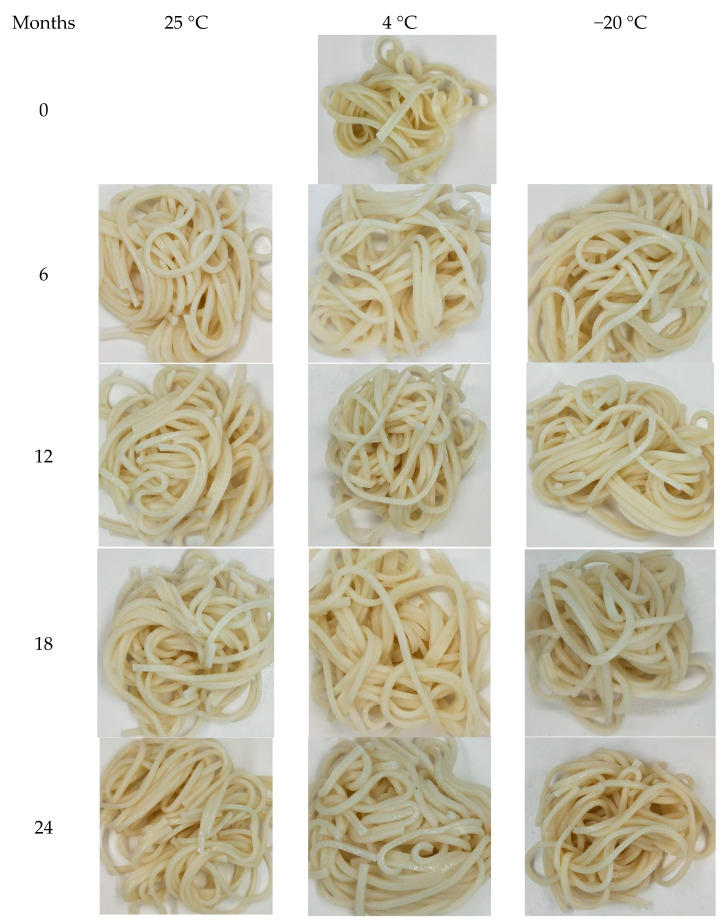
Appearance (after cooking) of cooked wheat noodles fortified with isomaltodextrin during different storage periods at different storage temperatures.

The impact of color characteristics on wheat noodles fortified with isomaltodextrin during different storage periods is in Table 2. The appearance of cooked wheat noodles fortified with isomaltodextrin for 0, 6, 12, 18, and 24 months of storage at 25, 4, and −20 °C is in Figure 3. Color parameters of L*, a*, and b* of noodle samples stored at 25 °C showed a significant deviation from 0 to 24 months. For noodle samples stored at 4 °C, the L* and a* value started to change beyond 9 months of storage. The a^*^ and b^*^ values of noodle samples stored at −20 °C did not differ; only the L* value differed after 6 months. Fortification of noodle samples with isomaltodextrin may alter the microstructure of the noodles, causing a disrupted starch, protein, and dietary fiber matrix. Changing the microstructure of the noodle samples may trigger a chemical reaction, such as browning effects and oxidation. Mohamed, Xu, and Singh (2010) [30] found that the Maillard reaction occurring in between reducing sugar and proteins may lead to the lightlessness of the cooked noodles. Color deterioration of food products during storage is mainly due to an enzymatic or chemical reaction [31]. Furthermore, during storage, the moisture is absorbed, and an oxidation reaction may also be responsible in part for the changes in color value [6]. Results of this color parameter study revealed that storage condition and duration of storage has a corresponding effect on the color of the noodle samples.

### 3.4. Effects of Storage on Texture Profile

Sensory attributes of fortified noodles rely on cooking qualities and texture of the cooked products, which also play an important role in affecting the acceptability of consumers. The texture profile of the cooked wheat noodles fortified with isomaltodextrin during different storage periods and temperatures is in Table 3. The hardness of the noodle samples stored at 25, 4, and −20 °C increased greatly from 4164.02 ± 49.37, 4172.81 ± 47.29, and 4189.19 ± 81.31 to 4271.44 ± 29.7, 4211.14 ± 53.34, and 4235.07 ± 47.84, respectively, at 24 months of storage. At 25 °C storage, the adhesiveness, maximum tensile strength, and tensile fracture distance significantly differed after 6, 12, and 9 months of storage, respectively. However, noodles stored at 4 and −20 °C showed significant differences for only hardness and adhesiveness at 24 months of storage period. The increase in adhesiveness of isomaltodextrin noodles during storage may be due to the increase in cooking loss encouraging disintegration of more soluble proteins and starch during cooking. In contrast, the partially soluble proteins and starch are attached on the surface of noodles, causing aggravation of this sticky behavior [32]. The increase in hardness and adhesiveness of the isomaltodextrin noodles indicated the deterioration of the noodle samples during storage, especially at 25 °C storage. The texture quality change in the isomaltodextrin noodle samples at different storage temperatures and periods may be due to the fiber supplements disturbing the fiber–starch matrix within the microstructure of noodles [33]. 

### 3.5. Effects of Storage on Sensory Properties

**Figure 4:**
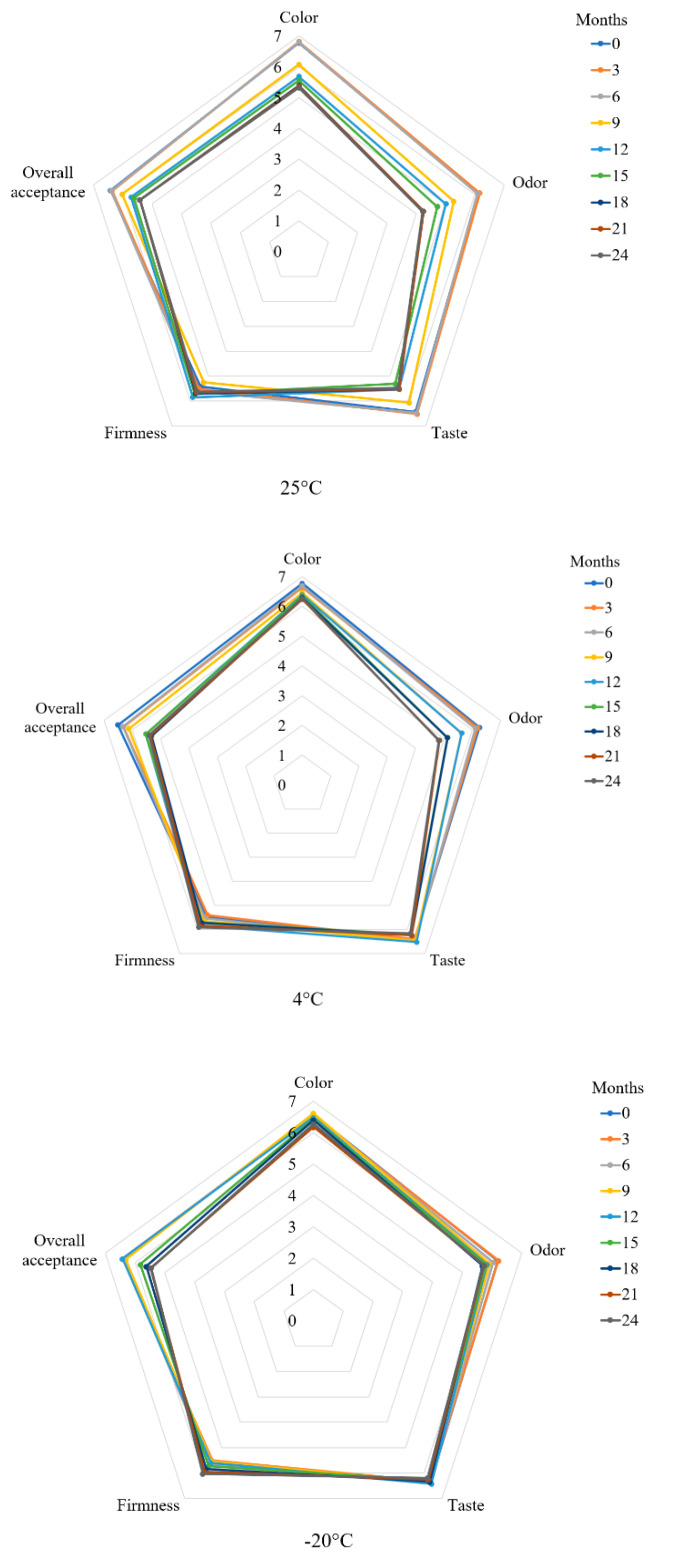
Sensory evaluation of wheat noodles fortified with isomaltodextrin during different storage periods at 25, 4, and −20 °C storage.

Figure 4 illustrates the sensory evaluation of wheat noodles fortified with isomaltodextrin during different storage periods (0, 3, 6, 9, 12, 15, 18, 21, and 24 months) at 25, 4, and −20 °C storage. The cooked isomaltodextrin noodles were evaluated for sensory characteristics such as color, odor, taste, firmness, and overall acceptance. Long-term storage reduced the overall acceptance of the noodle samples, which may due to the odor changed during the long-term storage. However, taste, firmness, and color did not differ for the isomaltodextrin noodles stored at −20 °C. Sensory attributes, including color, odor, taste, firmness, and even overall acceptance of the isomaltodextrin noodles stored at ambient temperature slowly deteriorated during the storage study, from 0 to 24 months. Even though the sensory attributes and overall acceptance of the isomaltodextrin noodles stored at 25, 4, and −20 °C was slightly reduced by time, all the samples were acceptable to the panelists, with a score >5. The firmness of the isomaltodextrin noodles was increased slightly for the noodles stored at 25, 4, and −20 °C, from 0 to 24 months, which may due to the loss of moisture and breakdown of sample matrix interconnection during the long-term storage and directly affects the cooking characteristics of the noodle samples [6].

### 3.6. Effects of Storage on Microstructural Characteristics

**Figure 5:**
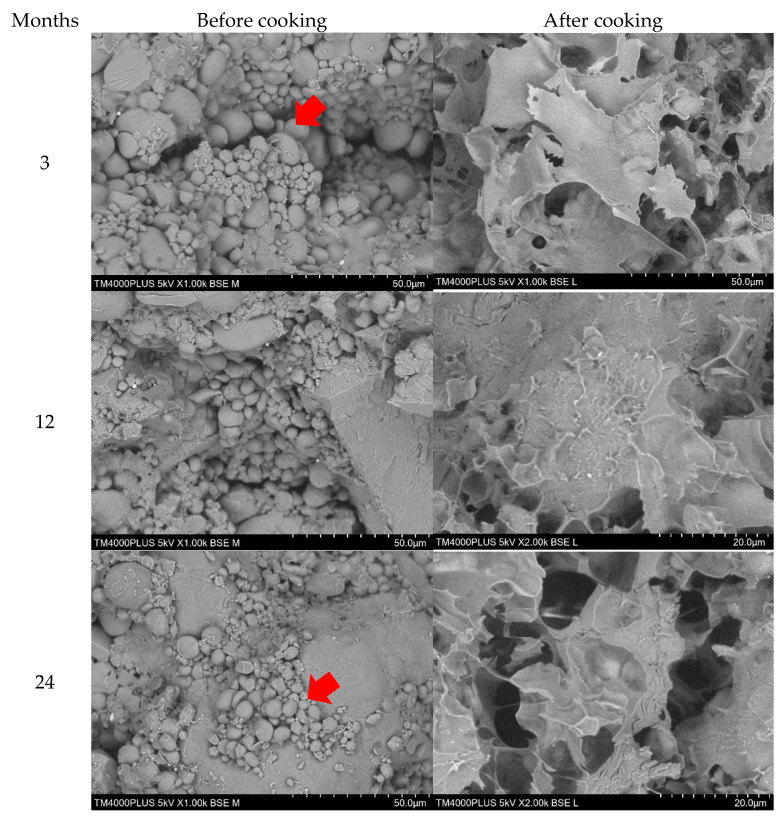
SEM of wheat noodles fortified with isomaltodextrin during different storage periods at 25 °C storage.

**Figure 6:**
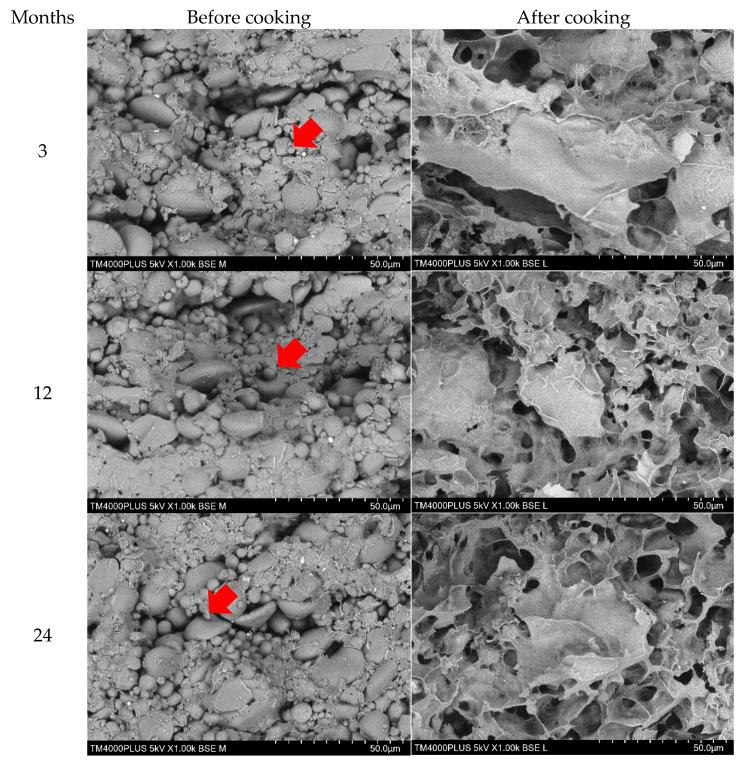
SEM of wheat noodles fortified with isomaltodextrin during different storage periods at 4 °C.

**Figure 7:**
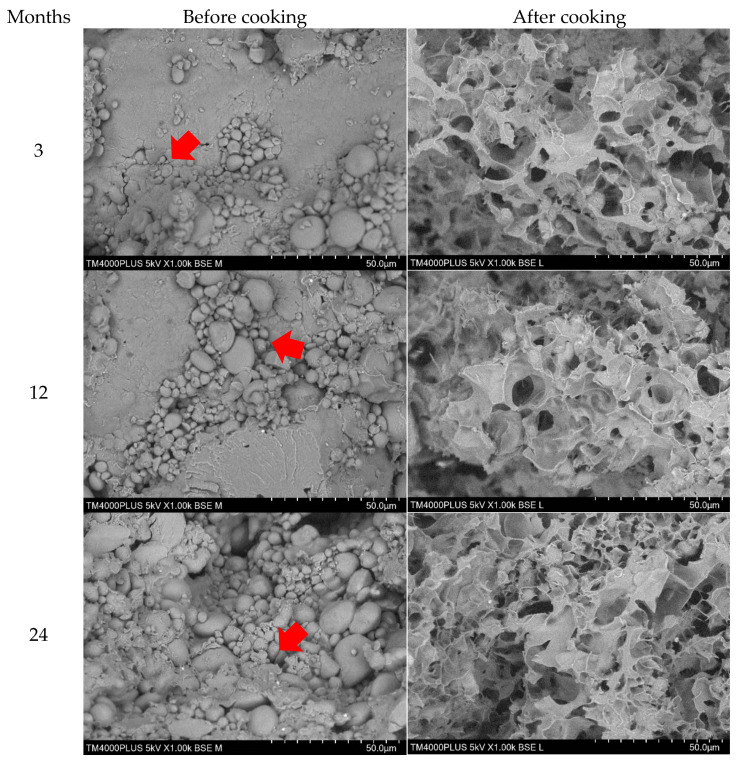
SEM of wheat noodles fortified with isomaltodextrin during different storage periods at −20 °C.

Scanning electron microscope (SEM) was used to collect data on the structural integrity, size, shape, and arrangement of particles of both raw and cooked wheat noodles fortified with isomaltodextrin, which closely correlated with the cooking quality, texture profile, and sensory characteristics [34]. Figure 5, Figure 6 and Figure 7 show the SEM micrographs of wheat noodles fortified with isomaltodextrin during different storage periods at 25, 4, and −20 °C, respectively. Micrographs of the raw isomaltodextrin noodle samples (before cooking) at 25 °C showed the well-formed fiber–starch matrix, with clearly seen isomaltodextrin particles attached to the starch granules. At 4 °C storage, the isomaltodextrin particles were partially adhered between each molecule and with the starch granules. The isomaltodextrin particles were frosted and held within starch granules to form a compact fiber–starch network because of the low temperature (storage temperature −20 °C). The starch granules within the noodle samples seemed to be a little swollen and with irregular size and shape, possibly demonstrating a gelatinization that occurred during the extrusion process [34]. Storage period did not affect the microstructure of the wheat noodles fortified with isomaltodextrin. SEM micrographs of raw noodle samples with isomaltodextrin showed a higher inclusion rate of soluble fiber in the binding relationship between starch and fiber (isomaltodextrin), which altered the general structural complexity of the noodle system.

Gelatinization of starch granules within the cooked noodle samples seems to be integrated into a developed fiber matrix to form a compacted noodles structure. The isomaltodextrin disturbs the structure with starch granules within the noodle matrix, causing loss of the regular structure and triggering the gelatinization process during cooking. The disruption to the isomaltodextrin noodles structures significantly affected the overall cooking quality and texture profiles of the isomaltodextrin noodles, which is described in Table 1 and Table 3.

## 4. Conclusions

During a storage and stability study, food products may go through physical, chemical, and biochemical deterioration along with environmental condition, such as temperature and time range, which may cause changes in the product’s physical, chemical, quality, and sensory attributes. These changes affect the safety of products and consumer acceptability. Hence, the quality and characteristics of the food products must be evaluated in a storage stability study to sustain the product’s stability. In this study, during 24 months of storage at low temperatures (4 °C and −20 °C), cooking quality, fiber profile, and color did not differ from 0 months, with only minor differences in texture and sensory characteristics. Therefore, isomaltodextrin noodles could be stored at low temperature to preserve the product’s functionality and quality.

## Figures and Tables

**Table 1:** Cooking quality characteristics of wheat noodles fortified with isomaltodextrin during different storage periods and temperature.

Months	Temperature (°C)	Cooking Time (min)	Cooked Weight Gained (g)	Cooking Loss (%)
0	25	7.38 ± 0.05 ^c^	63.97 ± 0.60 ^ab^	3.71 ± 0.02 ^bc^
	4	7.34 ± 0.07 ^c^	64.12 ± 0.37 ^a^	3.79 ± 0.03 ^bc^
	−20	7.38 ± 0.06 ^c^	63.99 ± 0.36 ^ab^	3.87 ± 0.05 ^ab^
3	25	7.43 ± 0.07 ^bc^	63.58 ± 0.50 ^bc^	3.78 ± 0.04 ^bc^
	4	7.43 ± 0.06 ^bc^	64.16 ± 0.56 ^a^	3.75 ± 0.06 ^bc^
	−20	7.41 ± 0.09 ^bc^	63.93 ± 0.21 ^ab^	3.81 ± 0.05 ^bc^
6	25	7.50 ± 0.18 ^a^	63.93 ± 1.08 ^ab^	3.74 ± 0.09 ^bc^
	4	7.39 ± 0.07 ^c^	63.96 ± 0.26 ^ab^	3.81 ± 0.08 ^bc^
	−20	7.35 ± 0.04 ^c^	63.62 ± 0.62 ^bc^	3.77 ± 0.06 ^bc^
9	25	7.43 ± 0.04 ^bc^	64.19 ± 0.93 ^a^	3.79 ± 0.08 ^bc^
	4	7.47 ± 0.06 ^ab^	63.39 ± 0.01 ^bc^	3.85 ± 0.06 ^bc^
	−20	7.43 ± 0.02 ^bc^	64.11 ± 0.49 ^a^	3.86 ± 0.02 ^ab^
12	25	7.47 ± 0.09 ^a^	63.49 ± 0.08 ^bc^	3.89 ± 0.07 ^ab^
	4	7.45 ± 0.06 ^bc^	63.21 ± 0.06 ^c^	3.74 ± 0.04 ^bc^
	−20	7.46 ± 0.08 ^ab^	64.53 ± 0.70 ^a^	3.78 ± 0.04 ^bc^
15	25	7.44 ± 0.02 ^bc^	63.36 ± 0.26 ^bc^	3.93 ± 0.04 ^a^
	4	7.47 ± 0.15 ^a^	63.58 ± 0.42 ^bc^	3.88 ± 0.04 ^ab^
	−20	7.38 ± 0.05 ^c^	64.25 ± 0.84 ^a^	3.86 ± 0.04 ^ab^
18	25	7.55 ± 0.04 ^a^	62.62 ± 0.15 ^c^	3.98 ± 0.05 ^a^
	4	7.45 ± 0.05 ^bc^	63.72 ± 0.52 ^ab^	3.87 ± 0.11 ^ab^
	−20	7.45 ± 0.04 ^bc^	64.29 ± 0.84 ^a^	3.85 ± 0.02 ^bc^
21	25	7.39 ± 0.03 ^c^	62.56 ± 0.16 ^c^	4.08 ± 0.07 ^a^
	4	7.44 ± 0.03 ^bc^	63.55 ± 0.46 ^bc^	3.88 ± 0.07 ^ab^
	−20	7.42 ± 0.05 ^bc^	64.19 ± 0.87 ^a^	3.87 ± 0.05 ^ab^
24	25	7.36 ± 0.03 ^c^	62.37 ± 0.16 ^c^	4.13 ± 0.07 ^a^
	4	7.43 ± 0.01 ^bc^	63.85 ± 0.62 ^ab^	3.97 ± 0.05 ^a^
	−20	7.45 ± 0.08 ^bc^	63.54 ± 0.55 ^bc^	3.92 ± 0.03 ^a^

Scores are presented as mean ± SD of triplicate analysis. The lowercase letters indicated significant difference in each column (*p < 0.05*).

**Table 2:** Color characteristics of wheat noodles fortified with isomaltodextrin during different storage periods.

Months	Temperature (°C)	L*	a*	b*
0	25	53.90 ± 1.59 ^a^	−2.95 ± 0.08 ^b^	4.19 ± 0.07 ^c^
	4	53.22 ± 1.96 ^a^	−2.95 ± 0.09 ^b^	4.25 ± 0.08 ^bc^
	−20	53.18 ± 1.95 ^a^	−2.88 ± 0.13 ^ab^	4.36 ± 0.07 ^bc^
3	25	53.60 ± 3.09 ^a^	−2.94 ± 0.09 ^b^	4.20 ± 0.08 ^c^
	4	48.51 ± 3.78 ^bc^	−2.86 ± 0.06 ^ab^	4.27 ± 0.07 ^bc^
	−20	50.59 ± 1.84 ^a^	−2.78 ± 0.08 ^a^	4.44 ± 0.09 ^bc^
6	25	49.38 ± 0.89 ^bc^	−2.96 ± 0.11 ^b^	4.01 ± 0.58 ^c^
	4	50.40 ± 1.16 ^a^	−2.92 ± 0.11 ^b^	4.27 ± 0.07 ^bc^
	−20	49.87 ± 0.96 ^bc^	−2.74 ± 0.04 ^a^	4.52 ± 0.11 ^ab^
9	25	49.13 ± 0.92 ^bc^	−3.17 ± 0.09 ^c^	4.59 ± 0.08 ^ab^
	4	48.37 ± 0.44 ^bc^	−2.83 ± 0.07 ^a^	4.13 ± 0.11 ^c^
	−20	48.65 ± 0.67 ^bc^	−2.78 ± 0.07 ^a^	4.28 ± 0.09 ^bc^
12	25	47.60 ± 1.23 ^bc^	−3.25 ± 0.09 ^c^	4.78 ± 0.07 ^a^
	4	49.01 ± 0.29 ^bc^	−2.87 ± 0.07 ^ab^	4.30 ± 0.05 ^bc^
	−20	48.87 ± 0.57 ^bc^	−2.77 ± 0.03 ^a^	4.51 ± 0.11 ^ab^
15	25	46.46 ± 1.06 ^c^	−3.33 ± 0.07 ^c^	4.68 ± 0.05 ^a^
	4	48.65 ± 0.95 ^bc^	−2.89 ± 0.06 ^ab^	4.30 ± 0.03 ^bc^
	−20	48.23 ± 0.31 ^bc^	−2.81 ± 0.11 ^a^	4.51 ± 0.06 ^ab^
18	25	46.39 ± 1.11 ^c^	−3.38 ± 0.04 ^c^	4.82 ± 0.04 ^a^
	4	47.63 ± 0.95 ^bc^	−3.08 ± 0.08 ^b^	4.35 ± 0.06 ^bc^
	−20	47.96 ± 0.66 ^bc^	−2.83 ± 0.09 ^a^	4.34 ± 0.13 ^bc^
21	25	44.88 ± 1.25 ^c^	−3.46 ± 0.04 ^c^	4.93 ± 0.03 ^a^
	4	47.23 ± 0.22 ^bc^	−3.09 ± 0.08 ^b^	4.34 ± 0.05 ^bc^
	−20	47.29 ± 0.16 ^bc^	−2.83 ± 0.09 ^a^	4.26 ± 0.04 ^bc^
24	25	42.83 ± 0.71 ^c^	−3.52 ± 0.22 ^c^	5.12 ± 0.04 ^a^
	4	47.21 ± 0.21 ^bc^	−3.19 ± 0.08 ^c^	4.36 ± 0.15 ^bc^
	−20	47.12 ± 0.05 ^bc^	−2.74 ± 0.02 ^a^	4.34 ± 0.04 ^bc^

Scores are presented as mean ± SD of triplicate analysis. The lowercase letters indicated significant differences in each column (*p* < 0.05).

**Table 3:** Texture analysis of wheat noodles fortified with isomaltodextrin during different storage periods.

Months	Temperature (°C)	Hardness (g)	Adhesiveness (g/s)	Maximum Tensile Strength (g)	Tensile Fracture Distance (mm)
0	25	4164.02 ± 49.37 ^c^	77.36 ± 0.83 ^c^	41.99 ± 0.65 ^ab^	107.07 ± 2.66 ^a^
	4	4172.81 ± 47.29 ^bc^	76.78 ± 1.96 ^c^	42.03 ± 0.27 ^ab^	107.65 ± 1.18 ^a^
	−20	4189.19 ± 81.31 ^bc^	77.81 ± 0.31 ^bc^	42.81 ± 0.31 ^ab^	107.52 ± 1.22 ^a^
3	25	4145.84 ± 50.49 ^c^	77.79 ± 1.26 ^c^	37.62 ± 0.51 ^b^	101.17 ± 1.24 ^ab^
	4	4217.83 ± 9.86 ^a^	77.01 ± 1.55 ^c^	40.94 ± 0.22 ^ab^	101.29 ± 1.09 ^ab^
	−20	4210.43 ± 54.29 ^a^	79.20 ± 0.94 ^ab^	42.36 ± 0.75 ^ab^	105.52 ± 1.52 ^a^
6	25	4154.76 ± 15.41 ^c^	78.28 ± 1.08 ^bc^	35.70 ± 0.42 ^c^	94.82 ± 1.49 ^ab^
	4	4180.45 ± 44.59 ^bc^	77.59 ± 0.93 ^c^	39.66 ± 0.25 ^ab^	103.09 ± 1.49 ^ab^
	−20	4198.39 ± 5.35 ^bc^	76.98 ± 0.82 ^c^	41.76 ± 0.59 ^ab^	101.07 ± 0.76 ^ab^
9	25	4187.61 ± 15.02 ^bc^	77.91 ± 0.59 ^bc^	34.06 ± 0.16 ^c^	85.47 ± 1.25 ^c^
	4	4204.14 ± 6.53 ^bc^	76.09 ± 1.33 ^c^	39.11 ± 0.15 ^ab^	99.87 ± 0.24 ^ab^
	−20	4196.64 ± 18.46 ^bc^	77.97 ± 1.84 ^bc^	41.71 ± 0.44 ^ab^	99.61 ± 0.80 ^ab^
12	25	4173.11 ± 31.86 ^bc^	79.05 ± 1.23 ^ab^	31.57 ± 0.27 ^c^	80.28 ± 1.81 ^c^
	4	4221.64 ± 91.03 ^a^	76.61 ± 1.74 ^c^	38.53 ± 0.39 ^b^	98.44 ± 0.77 ^ab^
	−20	4190.13 ± 48.19 ^bc^	78.19 ± 0.97 ^bc^	41.54 ± 0.99 ^ab^	97.49 ± 1.02 ^ab^
15	25	4186.11 ± 62.61 ^bc^	79.31 ± 1.14 ^ab^	29.01 ± 0.67 ^c^	76.01 ± 1.54 ^c^
	4	4157.65 ± 36.65 ^c^	78.75 ± 0.45 ^ab^	38.43 ± 0.26 ^b^	97.46 ± 0.96 ^ab^
	−20	4176.12 ± 63.16 ^bc^	79.64 ± 0.56 ^ab^	41.49 ± 1.61 ^ab^	98.21 ± 0.93 ^ab^
18	25	4210.48 ± 11.73 ^a^	80.05 ± 1.52 ^a^	26.94 ± 0.21 ^c^	74.23 ± 0.94 ^c^
	4	4128.51 ± 5.76 ^c^	76.64 ± 1.28 ^c^	36.61 ± 0.27 ^b^	95.79 ± 0.44 ^ab^
	−20	4243.74 ± 35.85 ^a^	79.39 ± 0.82 ^ab^	40.32 ± 0.56 ^ab^	95.75 ± 2.04 ^ab^
21	25	4190.08 ± 61.47 ^bc^	80.86 ± 2.02 ^a^	23.64 ± 0.48 ^c^	70.19 ± 0.93 ^c^
	4	4197.26 ± 41.38 ^bc^	78.41 ± 0.72 ^bc^	35.62 ± 0.31 ^c^	94.59 ± 0.45 ^ab^
	−20	4188.53 ± 10.96 ^bc^	79.39 ± 0.82 ^ab^	39.80 ± 0.52 ^ab^	97.26 ± 0.88 ^ab^
24	25	4271.44 ± 29.71 ^a^	82.71 ± 3.46 ^a^	21.84 ± 0.27 ^c^	67.47 ± 0.94 ^c^
	4	4211.14 ± 53.34 ^a^	80.24 ± 1.06 ^a^	35.65 ± 0.21 ^c^	94.65 ± 0.48 ^ab^
	−20	4235.07 ± 47.84 ^a^	79.89 ± 0.51 ^a^	39.05 ± 0.66 ^ab^	95.58 ± 0.61 ^ab^

Scores are presented as mean ± SD of triplicate analysis. The lowercase letters indicated significant differences in each column (*p <* 0.05).

## Supplementary Materials

The following are available online at https://www.mdpi.com/2223-7747/10/3/578/s1, Figure S1. Storage stability study design of wheat noodles incorporated with isomaltodextrin, Table S1: Changes in indigestible dextrin (ID), resistant starch, IHMWDF, and SHMWDF of wheat noodles with maltodextrin during different storage period at 25 °C storage temperature, Table S2: Changes in indigestible dextrin (ID), resistant starch, IHMWDF, and SHMWDF of wheat noodles with maltodextrin during different storage period at 4 °C storage temperature. Table S3: Changes in indigestible dextrin (ID), resistant starch, IHMWDF, and SHMWDF of wheat noodles with maltodextrin during different storage period at −20 °C storage temperature. Table S4: Changes in indigestible dextrin (ID), resistant starch, IHMWDF, and SHMWDF of wheat noodles with maltodextrin during different storage period at 40 °C storage temperature.
