# Supplementary material for: Quality Evaluation, Storage Stability, and Sensory Characteristics of Wheat Noodles Incorporated with Isomaltodextrin
Source: Plants (Basel). 2021 Mar 18;10(3):578. doi: 10.3390/plants10030578 (PMC8003340; doi:10.3390/plants10030578)
Supplement: Supplementary file 1 [file plants-10-00578-s001.pdf]

Supplementary material

	Page
Figure S1. Storage stability study design of wheat noodles incorporated with isomaltodextrin.	3
Method S1: Determining resistant starch (RS)	4
Method S2: Determining isomaltodextrin (ID)	6
Method S3: Determining IHMWDF and SHMWDF	8
Method S4: Sensory evaluation	10
Table S1: Changes in indigestible dextrin (ID), resistant starch, IHMWDF, and SHMWDF of wheat noodles with maltodextrin during different storage periods at 25 °C storage temperature.	11
Table S2: Changes in indigestible dextrin (ID), resistant starch, IHMWDF, and SHMWDF of wheat noodles with maltodextrin during different storage periods at 4 °C storage temperature.	12
Table S3: Changes in indigestible dextrin (ID), resistant starch, IHMWDF, and SHMWDF of wheat noodles with maltodextrin during different storage periods at -20 °C storage temperature.	13
Table S4: Changes in indigestible dextrin (ID), resistant starch, IHMWDF, and SHMWDF of wheat noodles with maltodextrin during different storage periods at 40 °C storage temperature.	14

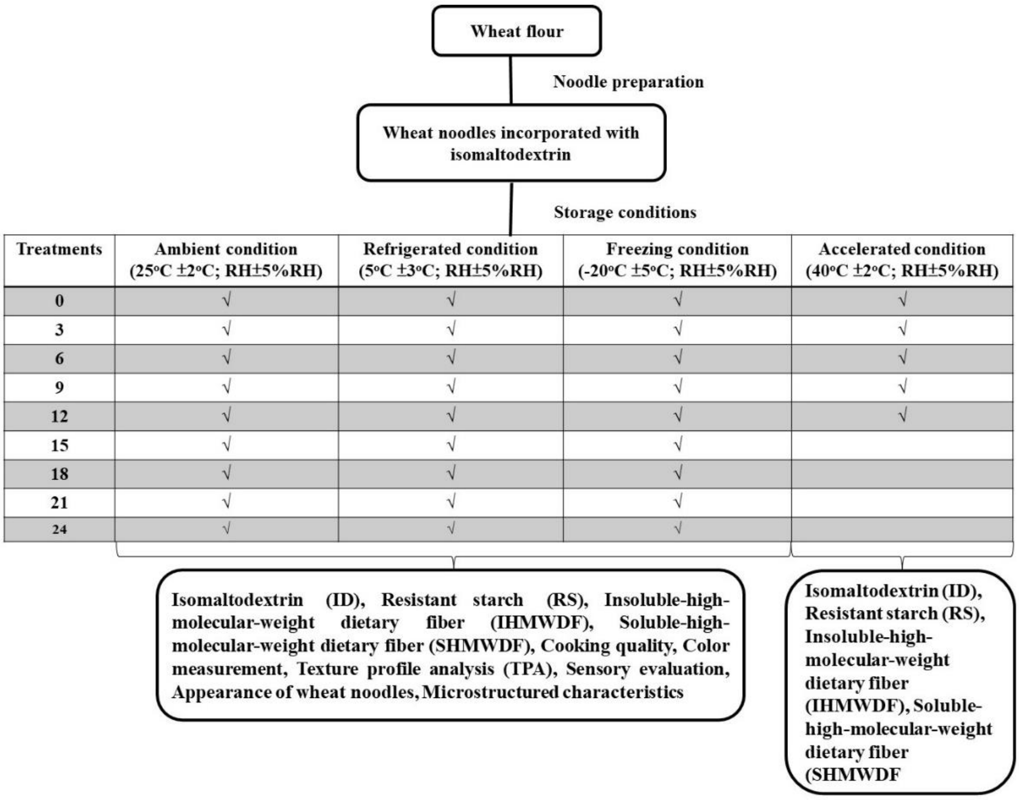

Treatments	Ambient condition (25°C ±2°C; RH±5%RH)	Refrigerated condition (5°C ±3°C; RH±5%RH)	Freezing condition (-20°C ±5°C; RH±5%RH)	Accelerated condition (40°C ±2°C; RH±5%RH)
0	√	√	√	√
3	√	√	√	√
6	√	√	√	√
9	√	√	√	√
12	√	√	√	√
15	√	√	√	
18	√	√	√	
21	√	√	√	
24	√	√	√	

Figure S1. Storage stability study design of wheat noodles incorporated with isomaltodextrin.

To understand the effect of storage conditions on the product quality, and by referring to the method of ICH Q1A (R2) Stability Testing of New Drug Substances and Products (European Medicines Agency), the noodle samples were stored under four different conditions, which is in general ambient condition (25 °C ± 2 °C/60 % RH ± 5 % RH), refrigerated storage temperature (5 °C ± 3 °C), freezing temperature (-20 °C ± 5 °C) for 24 months, and accelerated (40 °C ± 2 °C/75 % RH ± 5 % RH) conditions for 12 months.

Method S1: Determining resistant starch (RS)

A 100 mg amount of noodles minced into a powder sample was weighed accurately and directly put into a screw-cap polypropylene tube. A 4.0 mL amount of 10 mg/mL pancreatic α-amylase containing AMG (3 U/mL) was added into each tube, and the cap was closed tightly. The contents were mixed thoroughly by a vortex mixer and attached horizontally in a shaking water bath aligned in the direction of motion, in a shaking water bath set at 37 °C. The tubes were incubated at 37 °C with continuous shaking (200 strokes/min) overnight. The tubes were removed from the water bath one at a time, excess water on the surface was removed before opening the tube, and 4.0 mL ethanol (99% v/v) was added and continued with vigorous stirring on a vortex mixer. Next, tubes were centrifuged at 1500 g (approximately 3000 rpm) for 10 min without caps. Directly after the centrifuge was completed, the supernatants were decanted cautiously, and pellets re-suspended in 2 mL of 50% ethanol with vigorous stirring on a vortex mixer. A further 6 mL of 50% ethanol was further mixed into the tubes and centrifuged again at 1500 g for 10 min. The supernatant solutions were then carefully decanted and added to the original supernatant. The tubes containing the residue were upturned on absorbent paper to remove excess liquid while ensuring that the pellets were not extricated. A magnetic stirrer bar and 2 mL of 2 M KOH were added into each tube, and pellets were re-suspended (and the RS dissolved) by stirring for 20 min in an ice/water bath over a magnetic stirrer. An 8 mL amount of 1.2 M sodium acetate buffer (pH 3.8) was added to each tube with stirring on the magnetic stirrer. A 0.1 mL amount of AMG (3300 U/mL) was immediately added and mixed up thoroughly, the tubes were further incubated in a water bath at 50 °C for 30 min. For samples containing less than 10% RS content, the undiluted solutions were centrifuged aliquots at 8000 g for 5 min in a microfuge. A 0.1 mL amount of aliquots of the undiluted supernatants was transferred into glass test tubes, and 3.0 mL of glucose determination reagent (GOPOD reagent) was added and incubated at 50 °C for 20 min. The absorbance of each

solution was measured at wavelength 510 nm against the reagent blank. The RS content was calculated as follows:

Resistant Starch (g/100 g sample) (samples containing < 10% RS)

$= \Delta E \times F \times 10.3/0.1 \times 1/1000 \times 100/W \times 162/180$

$= \Delta E \times F/W \times 9.27$ --(S1)

where ΔE = absorbance (reaction) read against the reagent blank; F = conversion from absorbance to micrograms (the absorbance obtained for 100 μg of D-glucose in the GOPOD reaction is determined, and F = 100 (μg of D-glucose) divided by the GOPOD absorbance for this 100 μg of D-glucose; W = dry weight of sample analyzed.

Method S2: Determining isomaltodextrin (ID)

A 1.0 g amount of noodles minced powder sample was weighed accurately into a beaker; 40 mL of MES-TRIS buffer solution (pH 8.2) was added to each beaker with samples. A magnetic stirring bar was used to stir the mixture until the sample was completely dispersed in solution. Next, for incubating the sample with heat-stable α-amylase, while stirring, 50 μL of heat-stable α-amylase solution was added. Each single beaker was covered with aluminum foil and placed in a shaking water bath at 100 °C for 30 min with continuous agitation. All sample beakers were removed from the hot water bath and cooled to 60 °C after 30 min. A 10 mL amount of distilled water was used to rinse the side wall of the beaker, and a spatula was used to scrape other rings around the beaker and gels in the bottom of the beaker. A 100 μL amount of protease solution was added to each sample, which was covered with aluminum foil and incubated in shaking water bath at 60 °C for 30 min. A 5 mL amount of 0.56 N HCl solution was dispensed into sample while stirring. pH of the mixture was adjusted to pH 4.1–4.8 by 5% NaOH solution or 5% HCl solution. For incubating amyloglucosidase, 200 μL amyloglucosidase solution was added while stirring on a magnetic stirrer, followed by incubation in shaking water bath at 60 °C for 30 min.

To measure the total dietary fiber, 225 mL 95% ethanol pre-heated to 60 °C was added, and the ratio of ethanol volume to sample volume should be 4:1. All samples were covered with aluminum foil and allowed to stand at room temperature for 60 min for the precipitate formation. The precipitated enzyme digest from the sample was filtered through crucible. By using a vacuum, the residue was washed successively with two 15 mL portions of the 78% ethanol, 95% ethanol, and acetone. The crucible containing sample residue was dried overnight in an oven at 103 °C. The isomaltodextrin content was calculated as follows:

$$\text{isomaltodextrin (\%)} = \frac{\frac{R1+R2}{2} - p - A - B}{\frac{M1+M2}{2}} \times 100 \quad \text{---------------------------------------(S2)}$$

where R_1 = residue weight 1 from M_1; R_2 = residue weight 2 from M_2; M_1 = sample weight 1; M_2 = sample weight 2; A = ash weight from R_1; p = protein weight from R_2.

Method S3: Determining IHMWDF and SHMWDF

For the filtration setup, tare crucible containing 0.1 mg Celite was prepared. The bed of Celite in the crucible was wet and redistributed by using 15 mL of 78% (v/v) ethanol. Filtration was carried out by vacuum and filtering the enzyme digest through the crucible. A 21.9 mL amount of 60 °C deionized water was added to quantitatively transfer the remaining particles to crucible and rinsed residue. The combined filtrate was retained and transferred water was set aside for determining water SHMWDF. For IHMWDF, the residue was washed twice each with 15 mL of 78% ethanol, 95% ethanol, and acetone. The crucibles containing residue was dried overnight in a 105 °C oven. The crucible was cooled in a desiccator for approximately 2 h. The crucible containing IHMWDF residue and Celite was weighed to the nearest 0.1 mg. The residue from one crucible of the duplicates was analyzed for protein content by using the Kjeldahl method, and the second residue of the duplicates was analyzed for ash content by incineration overnight at 525 °C.

Each sample filtrated and transferred from IHMWDF was rinsed and added with water to bring the total volume to exactly 70 mL. Next, 279 mL of 60 °C of 95% of ethanol was added to mix thoroughly. The SHMWDF precipitate could form at room temperature for 60 min. The tare crucible containing 0.1 mg Celite was prepared as the filtration setup. The bed of Celite in the crucible was wet and redistribute by using 15 mL of 78% (v/v) ethanol. By using vacuum, the precipitated of SHMWDF was filtered from the supernatant through the crucible. An amount of 78% (v/v) ethanol was used to rinse and transfer all remaining particles to the crucible. The retain filtrate and washings was determining SHMWDF. The residue was washed twice each with 15 mL of 78% ethanol, 95% ethanol, and acetone. All the filtrates were combined. The crucibles containing residue were dried overnight in a 105 °C oven. The crucible was cooled down in a desiccator for approximately 2 h. The crucible containing SHMWDF residue and Celite was weighed to the nearest 0.1 mg. In the meantime, the residue from one crucible of the

duplicates was analyzed for protein content by using the Kjeldahl method, and the second residue was analyzed for ash content by incinerating overnight at 525 °C.

The IHMWDF and SHMWDF content was calculated as follows:

$$\text{IHMWDF (mg/100 g)} = \frac{\frac{R1+R2}{2} - p - A - B}{\frac{M1+M2}{2}} \times 100 \text{ -------------------------------------(S3)}$$

IHMWDF (%) = IHMWDF (mg/100 g)/1000

where R_1 = IHMWDF residue weight 1 from M_1; R_2 = IHMWDF residue weight 2 from M_2; M_1 = sample weight 1 in g; M_2 = sample weight 2 in g; A = ash weight from R_1; p = protein weight from R_2; B = blank.

$$\text{SHMWDF (mg/100 g)} = \frac{\frac{R1+R2}{2} - p - A - B}{\frac{M1+M2}{2}} \times 100 \text{ -------------------------------------(S4)}$$

SHMWDF (%) = SHMWDF (mg/100 g)/1000

where R_1 = SHMWDF residue weight 1 from M_1; R_2 = SHMWDF residue weight 2 from M_2; M_1 = sample weight 1 in g; M_2 = sample weight 2 in g; A = ash weight from R_1; p = protein weight from R_2; B = blank.

Method S4: Sensory evaluation

The test evaluated the sensory properties of the wheat noodles fortified with isomaltodextrin during different storage conditions and periods in terms of color, odor, taste, firmness, and overall acceptance. The panelists involved in this sensory evaluation test were trained in the identification and rating scales for the intensity of each attribute before testing. The panelists trained in the requirements and evaluation procedures before the test. Additionally, the correct and suitable terminology was used, and the scoring technique was taught. The panelists who were insensitive and underperformed were rejected during the training section. The noodle samples stored under three different conditions, 25, 4, -20 °C for 24 months were prepared, and the sensory evaluation test was conducted for every 3 months of storage stability condition. The randomly selected samples were placed on white, disposable polyform plates and covered with food wrap until testing. Each sample was randomly marked with a three-digit number. The panelists were required to rinse their mouth thoroughly with purified water in between testing different noodle samples. The wheat noodles fortified with isomaltodextrin were evaluated by quantitative descriptive analysis involving 11-point interval scale with scores from 0 to 10 for each attribute, 0 indicating no value and 10 indicating extremely strong value.

Table S1: Changes in indigestible dextrin (ID), resistant starch, IHMWDF, and SHMWDF of wheat noodles with maltodextrin during different storage periods at 25 °C storage temperature.

Months	I.D.	R.S.	IHMWDF	SHMWDF
0	9.47 ± 0.06^{a}	0.24 ± 0.01^{a}	7.96 ± 0.04^{a}	0.95 ± 0.02^{a}
3	9.42 ± 0.08^{ab}	0.24 ± 0.01^{a}	7.87 ± 0.02^{ab}	0.96 ± 0.04^{a}
6	9.46 ± 0.04^{a}	0.23 ± 0.01^{a}	7.87 ± 0.05^{ab}	0.93 ± 0.01^{ab}
9	9.43 ± 0.06^{ab}	0.23 ± 0.03^{a}	7.81 ± 0.03^{b}	0.88 ± 0.01^{b}
12	9.43 ± 0.01^{ab}	0.23 ± 0.01^{a}	7.68 ± 0.01^{c}	0.82 ± 0.01^{bc}
15	9.43 ± 0.04^{ab}	0.24 ± 0.01^{a}	7.67 ± 0.06^{c}	0.79 ± 0.01^{c}
18	9.39 ± 0.04^{ab}	0.24 ± 0.02^{a}	7.63 ± 0.02^{cd}	0.71 ± 0.03^{d}
21	9.35 ± 0.04^{ab}	0.24 ± 0.02^{a}	7.52 ± 0.02^{d}	0.65 ± 0.02^{e}
24	9.31 ± 0.03^{b}	0.22 ± 0.02^{a}	7.44 ± 0.04^{d}	0.61 ± 0.02^{e}

Scores are presented as mean ± SD of triplicate analysis. The lowercase letters indicated significant difference in each column ($p<0.05$). ID, isomaltodextrin; RS, resistant starch; IHMWDF, insoluble high-molecular-weight dietary fiber; SHMWDF, soluble high-molecular-weight dietary fiber.

Table S2: Changes in indigestible dextrin (ID), resistant starch, IHMWDF, and SHMWDF of wheat noodles with maltodextrin during different storage periods at 4 °C storage temperature.

Months	I.D.	R.S.	IHMWDF	SHMWDF
0	9.31 ± 0.01^{a}	0.26 ± 0.01^{a}	8.02 ± 0.04^{a}	1.01 ± 0.05^{a}
3	9.32 ± 0.03^{a}	0.27 ± 0.01^{a}	8.07 ± 0.13^{a}	0.98 ± 0.04^{ab}
6	9.28 ± 0.03^{a}	0.26 ± 0.01^{a}	8.04 ± 0.03^{a}	0.99 ± 0.07^{ab}
9	9.31 ± 0.09^{a}	0.27 ± 0.03^{a}	8.03 ± 0.03^{a}	0.94 ± 0.03^{ab}
12	9.35 ± 0.04^{a}	0.25 ± 0.01^{a}	7.95 ± 0.02^{ab}	0.91 ± 0.02^{b}
15	9.37 ± 0.04^{a}	0.26 ± 0.01^{a}	7.87 ± 0.02^{b}	0.88 ± 0.02^{bc}
18	9.36 ± 0.04^{a}	0.27 ± 0.01^{a}	7.82 ± 0.03^{bc}	0.86 ± 0.02^{bc}
21	9.29 ± 0.03^{a}	0.25 ± 0.01^{a}	7.76 ± 0.04^{bc}	0.84 ± 0.02^{bc}
24	9.31 ± 0.02^{a}	0.25 ± 0.02^{a}	7.68 ± 0.01^{c}	0.79 ± 0.03^{c}

Scores are presented as mean ± SD of triplicate analysis. The lowercase letters indicated significant difference in each column ($p<0.05$). ID, isomaltodextrin; RS, resistant starch; IHMWDF, insoluble high-molecular-weight dietary fiber; SHMWDF, soluble high-molecular-weight dietary fiber.

Table S3: Changes in indigestible dextrin (ID), resistant starch, IHMWDF, and SHMWDF of wheat noodles with maltodextrin during different storage periods at -20 °C storage temperature.

Months	I.D.	R.S.	IHMWDF	SHMWDF
0	9.32±0.03[b]	0.25±0.01[a]	7.89±0.04[a]	1.01±0.05[a]
3	9.36±0.02[ab]	0.25±0.01[a]	7.90±0.04[a]	0.98±0.04[a]
6	9.42±0.03[a]	0.25±0.01[a]	7.90±0.03[a]	0.99±0.07[a]
9	9.42±0.02[a]	0.26±0.01[a]	7.91±0.03[a]	0.94±0.03[a]
12	9.38±0.02[ab]	0.24±0.02[a]	7.84±0.07[ab]	0.91±0.02[ab]
15	9.40±0.04[ab]	0.26±0.01[a]	7.85±0.01[ab]	0.88±0.02[ab]
18	9.32±0.03[b]	0.25±0.02[a]	7.76±0.03[b]	0.86±0.02[b]
21	9.34±0.03[b]	0.24±0.01[a]	7.76±0.01[b]	0.84±0.02[b]
24	9.28±0.01[b]	0.26±0.02[a]	7.74±0.01[b]	0.79±0.03[b]

Scores are presented as mean ± SD of triplicate analysis. The lowercase letters indicated significant difference in each column ($p<0.05$). ID, isomaltodextrin; RS, resistant starch; IHMWDF, insoluble high-molecular-weight dietary fiber; SHMWDF, soluble high-molecular-weight dietary fiber.

Table S4: Changes in indigestible dextrin (ID), resistant starch, IHMWDF, and SHMWDF of wheat noodles with maltodextrin during different storage periods at 40 °C storage temperature.

Months	I.D.	R.S.	IHMWDF	SHMWDF
0	9.46±0.05^{a}	0.27±0.01^{bc}	7.96±0.01^{a}	0.96±0.07^{a}
3	9.34±0.04^{ab}	0.26±0.01^{c}	7.92±0.06^{a}	0.86±0.01^{ab}
6	9.30±0.03^{b}	0.25±0.02^{c}	7.75±0.04^{b}	0.77±0.02^{b}
9	9.24±0.05^{b}	0.28±0.01^{b}	7.62±0.01^{bc}	0.64±0.04^{c}
12	9.09±0.07^{c}	0.30±0.02^{a}	7.56±0.01^{c}	0.61±0.03^{c}

Scores are presented as mean ± SD of triplicate analysis. The lowercase letters indicated significant difference in each column ($p<0.05$). ID, isomaltodextrin; RS, resistant starch; IHMWDF, insoluble high-molecular-weight dietary fiber; SHMWDF, soluble high-molecular-weight dietary fiber.
